# Users’ Perspectives of Direct-to-Consumer Telemedicine Services: Survey Study

**DOI:** 10.2196/68619

**Published:** 2025-02-03

**Authors:** Kate Churruca, Darran Foo, Andrew Turner, Emily Crameri, Maree Saba, Samantha Spanos, Matthew Vickers, Jeffrey Braithwaite, Louise A Ellis

**Affiliations:** 1 Australian Institute of Health Innovation, Macquarie University, Macquarie Park, Australia; 2 Healthdirect Australia Limited, Sydney, Australia; 3 Eucalyptus Pty Ltd

**Keywords:** telemedicine, digital health technology, direct-to-consumer, digital tools, telehealth, consumer experience

## Abstract

**Background:**

Commercially run direct-to-consumer (DTC) telemedicine services are on the rise in countries such as Australia and the United States. These include DTC services that are web-based, largely asynchronous, and offer targeted treatment pathways for specific health issues (eg, weight loss or sexual function). It has been argued that DTC telemedicine improves access to health care and promotes patient empowerment. Despite research examining quality and safety issues, little is known about users’ reasons for accessing DTC telemedicine services or their perceptions of them.

**Objective:**

In this study, we aimed to examine the perspectives of Australian users accessing DTC telemedicine services, including the reasons for use, perceived benefits, and concerns, in addition to their usage and interaction with traditional general practice services.

**Methods:**

A web-based cross-sectional survey including questions on demographics, published and validated scales, and author-developed closed- and open-response questions was administered via REDCap in 2023 to Australian adults accessing DTC telemedicine services.

**Results:**

Among the 151 respondents, most (136/151, 90.1%) had seen a general practitioner (GP) in the previous 12 months and were somewhat or very satisfied (118/136, 86.8%) with the care, just over half found it easy to get an appointment with their GP (76/151, 50.3%), and a quarter found it difficult (38/151, 25.2%). Among the 136 respondents who had seen a GP, more than half either “never” (55/136, 40.4%) or “rarely” (23/136, 16.9%) discussed the information and treatment received from DTC telemedicine service with their GP. The majority of respondents were using a DTC telemedicine service offering prescription skin care (92/151, 60.9%), had received treatment in the previous 6 months (123/151, 81.5%), and had self-initiated care (128/151, 84.8%). The most frequently cited reasons for using DTC telemedicine were related to convenience (97/121, 80.2%) and flexibility (71/121, 58.7%), while approximately a third of the sample selected that it was difficult to see traditional health care provider in their preferred time frame (44/121, 36.4%) and that the use of DTC telemedicine allowed them to gain access to services otherwise unavailable through traditional health care (39/121, 32.2%). Most participants felt “more in control” (106/128, 82.9%) and “in charge” of their health concern (102/130, 78.5%) when using DTC telemedicine services, in addition to having “more correct knowledge” (92/128, 71.9%) and “feeling better informed as a patient” (94/131, 71.8%). “Costs of services” (40/115, 34.8%) and “privacy” (31/115, 27%) were the most frequently reported concerns with using digital health care technologies such as DTC telemedicine.

**Conclusions:**

We report that most users perceive DTC telemedicine services as offering ease of access and convenience, and that their use contributes to a greater sense of empowerment over their health. Concerns were related to data privacy and the costs of utilizing the services. Responses suggest that DTC telemedicine may be tapping into a previously unmet need, rather than complementing traditional health care provided by a GP.

## Introduction

Telemedicine services involve the provision of health care, including the assessment of conditions and the delivery of treatments, through telephone and other digital channels [1,2]. In the past decade, direct-to-consumer (DTC) telemedicine services provided by private companies have become increasingly commonplace; prominent early examples include *Teladoc,* which had 6 million members in the United States by 2013 [3], and now operates in 16 countries including Australia [4]. Consumers using DTC services are able to initiate access to care through the internet, without referral, and often with no prior relationship with the consulting clinician, an approach also sometimes labeled as “commercial virtual visits” [5,6]. As a business model, users are either charged directly, or visits are paid for by contracts between the DTC telemedicine service and health care insurers [7].

The benefits of DTC telemedicine services include convenience and efficiency [8,9]. Patients have reported relatively high levels of satisfaction in using these services [10] and, particularly in the United States, these services have been suggested to increase access to health care for those who do not have insurance coverage [3,5]. By providing consumers with ready access to information, advice, diagnoses, and treatments, DTC telemedicine arguably contributes to patient empowerment through greater knowledge or a sense of control over their health [11,12]. However, issues have been raised about the lack of integration with traditional health care services [3,8] and concerns about the quality of care provided. For example, Schoenfeld et al [5] conducted an audit of 8 of the most widely used DTC companies in the United States, finding that complete history-taking occurred only in about 70% of visits and that wrong or no diagnosis was provided in almost a quarter, with considerable variability identified between conditions (eg, urinary tract infection or low back pain) and companies. The appropriate use of antibiotics for pediatric patients was also found to be lower in DTC telemedicine service, compared with urgent care and primary care providers [13]. However, other recent studies have suggested comparable quality and safety to traditional services in the rate of side effects from medication to treat erectile dysfunction [14] and in the management of respiratory infections [15].

In Australia, the prevalence of DTC telemedicine services has grown rapidly since the COVID-19 pandemic [16]. This includes a new model of DTC telemedicine service, one that is largely asynchronous, may be paid for by consumers on a subscription basis, and is organized around providing medication or treatments for a particular condition or type of issue (eg, men’s health, sexual and reproductive health, skin conditions, and weight loss) [8,9,16]. In 2023, Foo and colleagues [16] identified more than 20 DTC telemedicine services in Australia that offer these “targeted treatment pathways for a specific issue or condition,” including *Moshy* [17], which markets skin care and weight loss services to women, and *Pilot* [18], which provides a range of men-focused treatments for conditions including weight loss, hair and skin issues, and sexual health issues. As the delivery of health care via DTC telemedicine platforms frequently emphasizes discrete medical conditions, it precludes the development of sustained therapeutic relationships between clinicians and patients. This modality stands in marked contrast to conventional primary care practice, wherein the longitudinal physician-patient relationship and continuity of care constitute fundamental pillars of clinical excellence. Traditional primary care practitioners employ a holistic approach to patient management, rather than compartmentalizing health concerns into isolated conditions—a paradigm that enables comprehensive health oversight and integrated care delivery.

Despite the rise of DTC telemedicine services in Australia, and in countries such as the United States, the viewpoints of consumers have been underexamined [16,19], and this is particularly true for new models of DTC telemedicine that offer targeted treatment pathways for specific health issues. A recently published scoping review identified a clear gap in understanding user perspectives, including the reasons patients may choose to use these services [20]; little is known about the benefits they perceive, and any reservations they have in accessing this type of service [8]. This study aimed to examine the perspectives of consumers accessing DTC telemedicine for specific issues, including interactions with traditional health care services, the types of DTC telemedicine services used and reasons for using, and perceived benefits and concerns.

## Methods

A web-based cross-sectional survey that included questions on demographics, published and validated scales, and author-developed closed- and open-response questions was administered to Australian adults accessing DTC telemedicine services.

### Participants and Recruitment

Convenience sampling of users of DTC telemedicine services was used. Participants were recruited through an Australian digital health care company offering multiple DTC telemedicine services organized around targeted treatment pathways for specific conditions [16]. The company is a leading provider of DTC telemedicine services in Australia, with recent expansion internationally. At the time of data collection, the 5 different services it ran broadly dealt with the following areas: (1) Women’s health care focusing largely on weight management and menopause, (2) Men’s health care, (3) Skin care, (4) Women’s fertility, and (5) Sexual well-being. The company moderates closed Facebook groups for users of these different services to interact with one another, share their journeys, and ask questions (medical advice is not permitted in the Facebook groups). An advertisement to take part in the survey was posted on the company’s closed Facebook communities in November 2023 seeking the perspectives of Australian adults on their use of DTC services.

### Materials

The web-based survey was conducted via REDCap hosted by Macquarie University. The survey included questions on traditional health care utilization, followed by a series of closed- and open-response questions about their use of DTC services, including reasons for using and their perceived benefits and concerns (see Multimedia Appendix 1). Finally, the survey asked for demographic information. All questions were optional to complete.

To assess reasons for using DTC telemedicine services, respondents were asked a series of closed-response questions about experiences with traditional health care services, including whether they had seen a general practitioner (GP) over the preceding 12 months, reasons for accessing traditional health care, satisfaction with this care, and ease of getting an appointment. They were also asked about how they had come to initiate the use of the DTC telemedicine service (ie, recommended by a health care provider, recommended by a family member or friend, or self-initiated) and their main reason(s) for using the service from a list of possible reasons derived from a literature review [3,5-9,16], which included, for example, challenges with accessing traditional health care and convenience.

To assess the perceived benefits of DTC services, we adapted the empowering outcomes scales from van Uden-Kraan et al [12] All 4 items from “being better informed” were used (eg, “I feel better informed as a patient”). From their “increased optimism and control” subscale, 5 of the 8 original items were used (eg, “I feel more in charge of the course of my health concern/s”). Minor modifications were made to wording, such as changing “illness” to “health concern/s.” Items for these subscales are answered on a 5-point Likert (1=completely disagree to 5=completely agree). To assess concerns about using digital health care technologies, participants were asked to select as many as apply from a range of options, derived from the literature [3,5-9,16], including safety and quality and the lack of regulation. Participants were also asked 2 open response questions, one about what they liked about using the DTC telemedicine service (ie, benefits), and one about their concerns with using digital health care technologies.

### Analysis

Closed-ended data were analyzed using descriptive statistics in IBM SPSS Statistics (version 29; IBM Corp.) to summarize patterns in experiences with traditional health care services, DTC telemedicine services accessed, reasons for using, perceived benefits, and concerns with using digital health care technologies. For location, postcode data were mapped to the Australian Statistical Geography Standard and classified as major city, regional area, or remote area. For the 2 open response questions, inductive content analysis [21] was conducted by KC in Microsoft Excel. This involved initial open coding to write short summaries (codes) of meaning units within each response; this was followed by identifying recurrent meaning units across responses to identify categories. Multiple categories could be present in the response for a single participant and were summarized by frequency and with example extracts.

### Ethical Considerations

The study received ethics approval from the Macquarie University Faculty of Medicine, Health and Human Sciences Low-Risk Ethics Subcommittee (No. 520221256844148). All participants opted in to participate and provided voluntary informed consent prior to completing the survey. The survey was designed to be anonymous, with no identifiable data collected. Those who took part in the survey had the option of going into the draw to win a $250 gift card.

## Results

Responses were received from 204 people; Table 1 summarizes the characteristics of the participants. A total of 151 (74.0%) of these responses included sufficient information for inclusion in the analysis; 113 respondents completed the demographic information. Approximately two-thirds of those responding to demographic questions (75/113, 66.4%) were female, most were between the ages of 30‐39 years (30/113, 26.8%) or 40‐49 years (32/113, 28.6%) and most were employed in full-time paid work (62/113, 55.4%).

Most participants rated their health as “good” (36/115, 31.3%) or “very good” (38/115, 33.0%). Approximately half (58/115, 50.4%) reported having at least one chronic condition, such as a mental or behavioral health condition, back problems, or asthma.

The DTC telemedicine services most frequently used were those for prescription skincare (92/151, 60.9%) and the men’s health care service (50/151, 33.1%); the women’s health care (4/151, 2.6%), fertility (6/151, 4%), and sexual well-being services (4/151, 2.6%) were each used by only a small number of participants. Some participants (13/151, 8.6%) reported accessing multiple services. Most respondents (123/151, 81.5%) had been prescribed treatment within the previous 6 months.

**Table 1.** Demographic characteristics of direct-to-consumer telemedicine users responding to the survey.

Characteristics	N (%)^a^
Sex	
Female	75 (66.4)
Male	38 (33.6)
Age, years	
18‐29	22 (19.6)
30‐39	30 (26.8)
40‐49	32 (28.6)
50‐59	13 (11.6)
>60	15 (13.4)
Highest level of education	
Postgraduate degree	21 (18.3)
Graduate diploma/certificate	18 (15.7)
Bachelor degree	26 (22.6)
Certificate level	27 (23.5)
Secondary School (year 12 or less)	21 (18.3)
Other	2 (1.3)
Employment status	
Full-time paid work	62 (55.4)
Part-time paid work	34 (30.4)
Unemployed	6 (5.4)
Retired	4 (3.6)
Other	6 (5.4)
Location	
Major city	71 (64.5)
Regional area	35 (31.8)
Remote area	4 (3.6)

a Valid percentage used. Note. Responses may not equal 151 owing to missing data.

### Experience With Traditional Health Care Services

Most participants (136/151, 90.1%) had seen a GP in the previous 12 months. Of the 15 respondents who had not, most (11/15, 73.3%) had not needed to; only 2 indicated it was too difficult to get an appointment, and 1 suggested it was too expensive. The most common reasons for seeing a GP were to get a prescription (67/136, 49.3%), management of an ongoing condition (64/136, 47.1%), a general or preventive health check-up (49/136, 35.0%), and for information/treatment of a new condition (46/136, 33.8%). A large majority of participants (118/136, 86.8%) were somewhat or very satisfied with the quality of care they received from their GP in the preceding 12 months. Approximately half found it easy or very easy to get an appointment with their GP (76/151, 50.3%), while a quarter found it difficult or very difficult (38/151, 25.2%), and the rest were neutral in their response. A majority of respondents either “never” (55/136, 40.4%) or “rarely” (23/136, 16.9%) discussed the information, advice, support, or treatment they received from the DTC telemedicine service with their GP; of the 136 respondents, 20 (14.7%) “sometimes” did and 22 (16.2%) either “often” or “always” talked to their GP.

### Reasons for Using DTC Telemedicine Services

Over four-fifths of respondents (128/151, 84.8%) indicated that they had self-initiated use of the DTC telemedicine service, while 16 of 151 (10.6%) reported that they had received a recommendation from a friend or family member; only 2 of 151 (1.3%) responded that their health care provider had recommended using the service. Reasons for accessing DTC telemedicine services are displayed in Table 2. The most frequently cited reasons were “convenience in accessing information, support, and treatment at a time and place of my choosing” (97/121, 80.2%), “the flexibility and choice available online” (71/121, 58.7%), and “difficulty in seeing a traditional health care provider in the timeframe I would like (eg, appointments booked out weeks in advance or times not suitable for me)” (44/121, 36.4%). Thirty-nine participants among 121 (32.2%) selected one of their main reasons as “gaining access to services or medications not available to me through traditional healthcare services.”

**Table 2.** Frequency of reported reasons for accessing direct-to-consumer (DTC) telemedicine services among users responding to the survey.

Reasons for accessing DTC telemedicine services	Number of respondents
Convenience in access at a time and place of my choosing	97
Flexibility and choice available online	71
Difficulty accessing traditional health care provider in the timeframe I would like	44
Gaining access to services/medications not available to me through traditional health care services	39
Costs associated with accessing traditional health care services (e.g., not eligible for Medicare, out-of-pocket fees)	39
Preference for accessing health care remotely at this time	31
Check whether my symptoms require attention by a traditional health care provider	16
Better understand the advice or treatment recommended by my traditional health care provider	10
Seeking a second opinion/verifying my traditional health care provider's advice	10
Other	6

### Perceived Benefits of Using DTC Telemedicine Services

**Figure 1.**
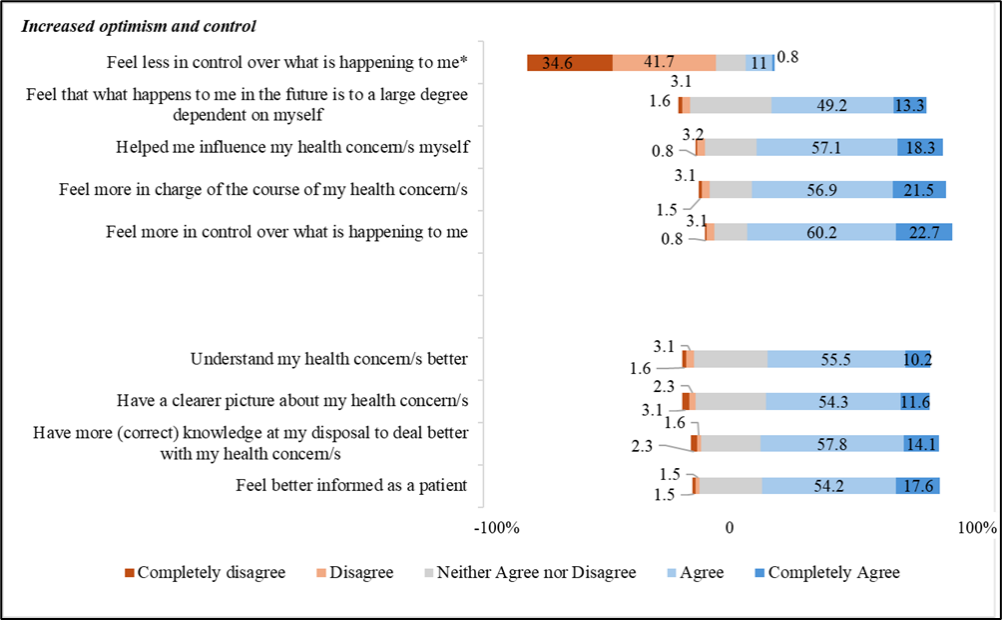
Perceptions of the impact of DTC telemedicine service use on feeling informed and having more control over health concern among users responding to the survey. * Item is negatively worded.

Figure 1 displays the frequency of participants’ responses to items on “being better informed” and having “increased optimism and control*”* from using the DTC telemedicine service. Most participants agreed that the information, advice, and support they received from the service had made them feel more in control over what was happening to them (106/128, 82.9%) and feel more in charge of the course of their health concern/s (102/130, 78.5%). Most disagreed that the use of the DTC telemedicine service had led them to feel less in control over what was happening to them (98/128, 76.6%). The majority of respondents agreed that they had more correct knowledge at their disposal to manage their health concern/s (92/128, 71.9%) and felt better informed as a patient (94/131, 71.8%).

Seventy-four participants (74/151; 49%) provided an open response for what they liked about using the DTC telemedicine service(s). Eight categories of benefits were identified (Table 3), with “ease and convenience” (n=46) being the most frequently cited.

**Table 3.** Categories of benefits of DTC telemedicine services identified from open response of users responding to the survey.

Category	Count	Exemplar quote(s)
Ease and convenience	46	“Ease of use, convenience” (ID132) “Convenient access at a time of my choosing” (ID188)
Quality of service	12	“Quality professional advice” (ID86) “Great communication from the staff (doctors)” (ID206)
Efficiency	11	“it means I don’t need to wait for an appointment to see my Dr” (ID119) “Ease of accessing treatments and to have it delivered with minimal downtime of face to face appointments” (ID81)
Benefits of the interface/platform	9	“It can be done from home and I am able to type messages in order to be better understood hopefully.” (ID145)
Privacy	8	“Some face-to-face appointments can be daunting either because of the cost associated or the topic of conversation itself. I like that it’s affordable, easily accessible and discreet” (ID110)
Cost savings	6	“It’s expensive and time consuming seeing a doctor face-to-face. (Service) works, and with little effort” (ID146)
Enabling access to medication/treatment	4	“It is a reliable way to get custom skin care with medical grade actives to help with acne control.” (ID197)
Greater control over health	3	“My ability to manage my needs myself to a degree.” (ID148)

### Concerns With Using Digital Health Care Technologies

Table 4 shows the frequency of participants’ concerns with using digital health care technologies. The most frequently selected concern was “the costs associated with some of these services” (40/115, 34.8%), followed by “concerns over privacy and what the information I provide will be used for” (31/115, 27%).

Thirty-four respondents (34/151, 22.5%) provided open response answers for their concerns about using digital health care technologies, which were coded into 6 categories. The most frequent category of concern was related to “privacy and data security” (n=13). Table 5 summarizes the categories of concerns with using digital health care technologies.

**Table 4.** Frequency of reported concerns with using digital health care technologies among direct-to-consumer telemedicine users responding to the survey.

Concerns with using digital health care technologies among DTC telemedicine users	Number of respondents
Costs associated with services	40
Concerns over privacy and what the information I provide will be used for	31
Lack of knowledge of services	27
Lack of integration	26
Concerns about safety, quality, or accuracy of the advice and services	25
Lack of regulation	17
Other	6

**Table 5.** Categories of concerns with using digital health care technologies identified from open response of direct-to-consumer telemedicine users responding to the survey.

Category	Count	Exemplar quote(s)
Privacy and data security	13	“Concerned about my data leaking (because I am getting treatment for Erectile Dysfunction)” (ID178) “Data being collected and sold” (ID38)
Lack of face-to-face interaction	6	“Them not being able to physically assess me” (ID71)
Expensive	6	“More so the costing as it can get expensive” (ID46)
Need to share with GP	3	“If I had a problem with treatment I would be best to see my GP about it” (ID36)
Lack of regulation	3	“Might be a scam” (ID164)
Quality issues	2	“Authenticity of the providers and the information given” (ID181)
Other	5	For example, difficulty accessing medication, digital literacy.

## Discussion

### Principal Results

This study of Australian users of new DTC telemedicine services, organized around specific health issues (eg, weight loss and skin concerns), demonstrated that users largely seek out these services of their own volition for reasons of convenience and flexibility. As a result of using these services, most users feel they are better informed and have greater control over their health concerns; they perceive further benefits in the ease of use and, to a lesser extent, the quality of the services. Users were less likely to indicate concerns with using digital health care technologies and, where they did, these mostly related to personal factors such as the costs of services or reservations about the privacy of personal health information.

### Comparison With Prior Work

Commentaries and empirical research have previously indicated that the use of DTC telemedicine services may support access to health care [3,10]. However, results from the present study do not indicate difficulty in accessing health care is a great driver of use, with most of the sample participants having seen a GP in the past year, reporting satisfaction with the service, and the ability to get an appointment. Prior research largely comes from the United States, which has a more pluralistic health care system with greater inequity in access than Australia. Moreover, these studies have predominately focused on DTC services providing synchronous “virtual visits” for urgent, but not life-threatening, conditions [3,5,10], rather than the new forms of DTC that offer “lifestyle medications” for the management of often ongoing health issues (eg, weight management, acne, and erectile dysfunction) [8,16]. For the latter service type, our results indicate that the benefit may be less in accessing health care than in accessing specific treatments that are otherwise inconvenient, difficult, or impossible to access through one’s traditional health care provider, even despite the increased adoption of telehealth in Australian general practice since the COVID-19 pandemic [22].

Very few respondents indicated that they used DTC telemedicine to complement traditional health care (such as getting second opinions or checking symptoms). This suggests DTC telemedicine may be tapping into previously unmet health care needs, rather than simply replacing conventional GP visits. Most respondents also rarely if ever discussed using these services with their GP, further implying the provision of care for their specific health condition remained separate to their use of traditional health care services. What is apparent, though, is that use of these services contributed to a sense of empowerment among participants, who indicated feeling better informed and able to manage their health condition, a finding also identified by Kyweluk [23] in a study of users of DTC ovarian reserve testing services.

Compared with these benefits, participants in this sample far less frequently highlighted concerns with using digital health care technologies, such as DTC telemedicine services. The most common apprehensions were related to personal issues of privacy and cost; few respondents indicated feeling concerned about more systemic factors such as lack of regulation, integration, and quality and safety, despite these being frequently raised in the literature [3,8,9]. The perception of these services as costly may be more pertinent to Australian consumers, many of whom have historically been able to visit a GP without incurring out-of-pocket costs [24], and then access heavily subsidized medicines through the Pharmaceutical Benefits Scheme [25].

### Strengths and Limitations

The use of existing subscales on patient empowerment [12], and the triangulation of qualitative and quantitative data analysis, are strengths of the study. Limitations include potential self-selection bias in responses to the survey. Data were missing for some demographic characteristics and other variables due to attrition and the fact that all responses were optional; we lack information as to the profile of users of these DTC telemedicine services for specific health conditions to understand how representative the sample may have been.

### Conclusions

This study provides insights into perspectives of patients who use DTC telemedicine services for specific health concerns, which is an emergent area of the health care system and one with limited research to date. Through a survey of users, benefits of these services were identified, including ease of access, convenience, and providing a sense of empowerment. Some concerns were identified related to data privacy and the costs of utilizing the services. Further research is needed to understand how the use of DTC telemedicine fits into the existing landscape of primary health care and the degree to which the use of these services provides a complement, supplement, or alternative to traditional general practice.

## Supplementary material

10.2196/68619 Multimedia Appendix 1 Survey items.

## Abbreviations

DTC: direct-to-consumer
GP: general practitioner
