# Supplementary material for: Users’ Perspectives of Direct-to-Consumer Telemedicine Services: Survey Study
Source: JMIR Form Res. 2025 Feb 3;9:e68619. doi: 10.2196/68619 (PMC11809934; doi:10.2196/68619)
Supplement: Multimedia Appendix 1 [file formative-v9-e68619-s001.docx]

**Multimedia Appendix 1. Survey items**

| **Construct** | **Question** | **Response options** |
| --- | --- | --- |
| Demographics | What is your gender? | - Female - Male - Other - Prefer not to answer |
|  | In what year were you born? | - [enter 4-digit birth year; eg. 1976] |
|  | What is the highest level of education that you have completed?  If you are currently studying, but have not completed a qualification, please answer with the highest level you have completed | - Postgraduate Degree - Graduate Diploma / Graduate Certificate - Bachelor Degree - Certificate level - Secondary school (Year 12 or less) - Other |
|  | What is your current work status? | - Full-time paid work (>35 hours per week) - Part-time paid work (< 35 hours per week) - Unemployed - Retired - Other |
|  | What is your postcode? | [enter 4-digit postcode] |
| Experience of traditional healthcare | Please indicate which of the following healthcare providers you have seen in the last 12 months. Select as many as apply | - A general practitioner (GP) - A specialist doctor (e.g., a cardiologist, surgeon, psychiatrist) - A mental health professional (e.g., counsellor or psychologist) - An allied health service provider (e.g., physiotherapist, occupational therapist) - A complementary or alternative therapies practitioner (e.g., acupuncture, naturopathy) - None of the above |
|  | For what reason(s) have you been to the GP in the previous 12 months? Select as many as apply | - Management for an ongoing condition - Information/treatment advice for a new condition or concern - Specifically, to get a prescription - To get a medical certificate - To get a referral to another healthcare provider - To get a referral for further tests or investigations - General or preventative health check-up - Vaccination |
|  | Overall, how satisfied are you with the quality of healthcare you have received from GP(s) in the last 12 months? | - Very satisfied - Somewhat satisfied - Neither satisfied nor dissatisfied - Somewhat dissatisfied - Very dissatisfied |
|  | Recent data from the Australian Bureau of Statistics suggests 83% of people have consulted with their GP at least once in the previous 12 months. Please indicate why you have not seen a GP in the last 12 months. Select all that apply | - I have not needed to (e.g., I am in great health) - I self-manage my health - I use online health information and treatments - Too difficult to get an appointment - Can't afford it / too expensive - Other (please specify) |
|  | How easy is it for you to get an appointment with your preferred GP when you need one? | - Very easy - Easy - Neutral - Difficult - Very difficult - Don't know Haven't tried |
|  | To what extent did you discuss the information, advice, support or treatment you received from [DTC telemedicine service] with a general practitioner (GP) | - Never - Rarely - Sometimes - Often - Always - Does not apply to me |
| Use of DTC Telemedicine services | What is your awareness and experience of using the following platforms?   1. [DTC telemedicine service for women’s healthcare - weight management and menopause] 2. [DTC telemedicine service for men’s healthcare] 3. [DTC telemedicine service for skin care] 4. [DTC telemedicine service for women’s fertility] 5. [DTC telemedicine service for sexual wellbeing] | - I am UNAWARE of this - I am AWARE of this, but have NOT USED it - I have USED this |
|  | When was the last time you were prescribed a treatment on the [DTC telemedicine service] platform? | - < 3 months ago - 3 - 6 months ago - 6 - 12 months ago - 1 - 2 years ago - 2 years ago |
| Reasons for using DTC telemedicine service | Did you initiate use of [DTC telemedicine services] or was it recommended by your healthcare provider? Select as many as apply | - Recommended by a healthcare provider - Recommended by a family member or friend - I decided to use it myself |
|  | What are your MAIN reasons for using a service on the Eucalyptus platform? Select as many as apply | - Difficulty in seeing a traditional healthcare provider in the timeframe I would like (e.g., appointments booked out weeks in advance, times not suitable for me) - Convenience in accessing information, support, and treatment at a time and place of my choosing - Costs associated with accessing traditional healthcare services (e.g., not eligible for Medicare, out-of-pocket fees) - The flexibility and choice available online - To gain access to services or medications not available to me through traditional healthcare services - To seek a second opinion or verify my traditional healthcare provider's advice - To check whether my symptoms require attention by a traditional healthcare provider - To better understand the advice or treatment recommended by my traditional healthcare provider Preference for accessing healthcare remotely at this time - Other (please explain) |
| Benefits of using DTC telemedicine service | Thinking about any [DTC telemedicine services] you have used, please indicate the extent to which you agree with the following statements. The information, advice and support I have received by my use of [DTC telemedicine service(s)] has helped me:   1. Feel better informed as a patient 2. Understand my health concern/s better 3. Have a clearer picture about my health concern/s 4. Have more (correct) knowledge at my disposal to deal better with my health concern/s 5. Feel more in charge of the course of my health concern/s 6. Influence my health concern/s myself 7. Feel more in control over what is happening to me 8. Feel less in control over what is happening to me 9. Feel that what happens to me in the future is to a large degree dependent on myself | - Completely disagree - Disagree - Neither agree nor disagree - Agree - Completely agree |
|  | Please let us know, in your own words, what you like about using services on the Eucalyptus ecosystem? | [Open response] |
| Issues or concerns | What are your MAIN issues or concerns about using digital healthcare technologies in general? Select as many as apply | - My lack of knowledge about the other similar digital services available - Concerns about the safety, quality or accuracy of the advice and services provided - Concerns over privacy and what the information I provide will be used for - The lack of regulation of these tools and services - The lack of integration of these services with the wider health system - The costs associated with some of these services - Other |
|  | Please let us know, in your own words, what your concerns are about using digital healthcare technologies in general. | [Open response] |
